# Annotations

**Published:** 1903-11-14

**Authors:** 


### ANNOTATIONS.

### The German Emperor.

Happily the recent operation which the German Emperor has undergone for the removal of a small tumour from one of the vocal cords has been attended with no evil results, and need not give rise to any alarm. Apparently Dr. Moritz Schmidt, who performed the operation, had formed the opinion previous to its removal that the growth was of a benign nature. This opinion has been shown to be correct by a subsequent examination of the tumour, the microscopical appearances of which prove it to be so. The growth consisted of a connective tissue base covered with a normal layer of stratified epithelium which was in regular layers, and was everywhere sharply distinct from the connective tissue. That considerable anxiety should have been manifested when the news of the operation was at first received is only natural in view of all the circumstances, and the public will be much relieved to know that there are now no grounds at all for any apprehensions with regard to the Emperor's complete recovery.

### Sleeping Sickness.

Although the report actually made to the Royal Society by the Commissioners appointed to investigate the nature and causes of sleeping sickness in Uganda will presumably be presented to the Government before it is published by official authority, enough has been made known of its nature to justify the belief that the disease has been definitely shown to be due to the presence in the blood and cerebrospinal fluid of a species of the protozoon known as trypanosoma, an organism which was first discovered by Gruby in the blood of frogs and other amphibians, and another variety of which has since been shown to be communicated to horses by the bite of the tsetse fly, *Glossina morsitans*, an enlarged model of which, as well as of the trypanosomata which it communicates, may be seen in the Natural History Museum at South Kensington. The agent in the diffusion of the trypanosoma of sleeping sickness is a fly of a somewhat different species from the tsetse, although of the same family, and is distinguished as *Glossina palpalis*; while it may be presumed that in time characteristics will be discovered by which its peculiar form of trypanosma will admit of separation from its kindred. The tsetse parasite, as is well known, is fatal to horses, oxen and dogs, but appears to be harmless to most other animals and to man; while it is a curious fact that the sleeping sickness, which has destroyed the lives of thousands of natives in Uganda and other parts of Africa, has not yet been known to attack a white man. The Commission has, however, succeeded in communicating sleeping sickness to monkeys by inoculation with blood containing the trypanosoma, and is said, generally speaking, to have solved all the questions concerning the nature and methods of diffusion of the disease. It follows that those relating to prevention and to cure are ripe for inquiry. It is of course possible that some agent capable of destroying the trypanosoma in the blood may be discovered; but the extermination of the *Glossina palpalis* itself in its breeding places is the proceeding that probably offers the greatest prospect of success, and the settlers in Uganda cannot fail to be encouraged in this endeavour by the good results which have attended analogous proceedings against the malaria-carrying anopheles. Like its relative the tsetse fly, the *Glossina palpalis* is probably of restricted range; and the task of tracing it to its haunts, and of systematically destroying its larvæ, may possibly be found not to present insuperable difficulties.

### The Sale of Poisons.

Whatever views may be held regarding the merits of free trade in other departments, no one in the present day proposes to apply the principle to the sale of poisons. It is indeed an accepted position, and one recognised by the law of the land, that the public interest and safety demand some measure of restriction in the sale and purchase of substances which may be possibly used to destroy human life. Concerning the extent of such restriction there is considerable difference of opinion, more especially among the various groups of traders. The commercial spirit very naturally seeks to maintain, or to destroy, the practice of legal restraint according as it happens to be within or without the protected area. With these trade squabbles the public has little concern so long as they are not permitted to disturb the conditions essential to the protection of peaceable citizens. These conditions are few in number. The first is that the purchase of poison shall be accomplished, not readily, but with such a degree of difficulty as to hinder rather than to facilitate the plans of the would-be evildoer. The second condition demands that the purchaser of poison shall complete the transaction in circumstances which will establish a clue to his identity should the event render this necessary. Given these two conditions reasonable men may sleep in peace. The existing regulations by which the sale of actively poisonous substances is restricted to pharmacists, work on the whole fairly well, and have on more than one occasion proved their value as aids in the detection of the criminal poisoner. Some attempts are at present being made to relax the severity of these regulations on the ground that they interfere with the ready purchase of various poisons used for manufacturing or similar purposes. Whether this contention is or is not well founded, it must be remembered that it is entirely secondary to the interest of the general public. And it is difficult to allow that this interest can be promoted by the easy distribution of arsenic and other powerful poisons by oilmen, drysalters, seedsmen, and the like. The difficulty is increased when it is proposed to permit these poisons to be sold in preparations bearing fanciful names innocent of all suggestion of their dangerous nature. So far from relaxing the present law, it would be better to apply its provisions to all preparations, however named, which contain the poisons scheduled in the Pharmacy Act. This would ensure the application of the two conditions already defined. It carries also the further advantage of restricting the distribution of poisons to those who, from their technical training, are competent to appreciate the responsibility which is entailed in the sale of these dangerous substances.

